# Supplementary material for: Chromothripsis during telomere crisis is independent of NHEJ, and consistent with a replicative origin
Source: Genome Res. 2019 May;29(5):737–49. doi: 10.1101/gr.240705.118 (PMC6499312; doi:10.1101/gr.240705.118)
Supplement: Supplemental Material [file supp_gr.240705.118_Supplemental_file_1.zip › contigs/annotated_contigs/DB57/contig.3.DB57_length_452_mean_cov_7.16150442478.docx]

**DB57_length_452_mean_cov_7.16150442478**

```
GCAAAAATATCAAAAATTAGCTGGGCATGGTGGCG|GG|CGCCTGTAGTCCCAGCTACTCGGGAGGCTGAGGCAGGAGAATCGCTTGAA
 >chr7:129305600-129305635 - E=2e-03 p=0e+00  >chr17:65271186-65271481 - E=7e-121 p=2e-
CCTGGGAGGCGGAGGTTGCAGTGAGCCGAGGTGCACCACTGCACTCCACCCTGGGCAACAGAGCAAGACCCTGTCTCAAATAAATACAT
02
AAATAAAATAAAATAAGTCTGGGCGTGGTGGCTGATGTCTGTAATCCCAGCACTTTGGGAGGCCAAGGCGGGCAGATCACCTGATATCA

GGAGTTCAAGACCAGCCTGACCAACATGGTAAAACCCCGTCTCTACTAAAAATACAAAAATTAGCTG|TATTCTAATT|ACAAGGTATA
                                                                              >chr17:67
AGTGTTCGTATTTCTCTGCAGCCTCACCAGCATCTATTGTTTTTTGACTTTTTAATAACGGCCATTCTGACGATTATGAGATGATATCT
769375-67769485 - E=1e-51
CATTGTGGTTT
```
